# Combined 3D Endoanal Ultrasound and Transperineal Ultrasound Improves the Detection of Anal Sphincter Defects †

**DOI:** 10.3390/diagnostics13040682

**Published:** 2023-02-11

**Authors:** Dan Carter, Edward Ram, Tal Engel

**Affiliations:** 1 Department of Gastroenterology, Chaim Sheba Medical Center, Tel Hasomer, Ramat Gan 5266202, Israel; 2 Sackler Faculty of Medicine, Tel Aviv University, Tel Aviv 6997801, Israel; 3 Department Surgery B, Chaim Sheba Medical Center, Ramat Gan 5266202, Israel

**Keywords:** fecal incontinence, perianal ultrasound, endoanal ultrasound, anal sphincter, sphincter injury

## Abstract

Introduction: Anal sphincter injury, mainly due to obstetric or iatrogenic etiology, is the most common cause of fecal incontinence (FI). Three-dimensional endoanal ultrasound (3D EAUS) is used for assessment of the integrity and the degree of anal muscle injury. However, 3D EAUS accuracy may be hampered by regional acoustic effects, such as intravaginal air. Therefore, our aim was to examine whether a combination of transperineal ultrasound (TPUS) and 3D EAUS would improve the accuracy of detection of anal sphincter injury. Methods: We prospectively performed 3D EAUS followed by TPUS in every patient evaluated for FI in our clinic between January 2020 and January 2021. The diagnosis of anal muscle defects was assessed in each ultrasound technique by two experienced observers that were blinded to each other’s assessments. Interobserver agreement for the results of the 3D EAUS and the TPUS exams was examined. A final diagnosis of anal sphincter defect was based on the results of both ultrasound methods. Discordant results were re-analyzed by the two ultrasonographers for a final consensus on the presence or absence of defects. Results: A total of 108 patients underwent ultrasonographic assessment due to FI (mean age 69 ± 13). Interobserver agreement for the diagnosis of tear on EAUS and TPUS was high (83%) with Cohen’s kappa of 0.62. EAUS confirmed anal muscle defects in 56 patients (52%), while TPUS confirmed them in 62 patients (57%). The final consensus agreed on the diagnosis of 63 (58%) muscular defects and 45 (42%) normal exams. The Cohen’s kappa coefficient of agreement between the results of the 3D EAUS and the final consensus was 0.63. Conclusions: The combination of 3D EAUS and TPUS improved the detection of anal muscular defects. The application of both techniques for the assessment of the anal integrity should be considered in every patient going through ultrasonographic assessment for anal muscular injury.

## 1. Introduction

Fecal incontinence (FI) is defined as the unintentional loss of solid or liquid feces, and it mirrors the final common path of multiple etiological factors. The reported prevalence of FI is 1.6–15% [1,2]. FI has a major negative impact on quality of life and daily activities [3], triggering physical and psychological morbidity, and it is often accompanied by severe social restriction [4]. Obstetric and iatrogenic injuries are the most common etiologies of anal sphincter injury [5,6], and damage to the anal sphincter is the most common cause of severe fecal incontinence (FI) [7]. The reported incidence of obstetric anal sphincter injury is 2.9% in the United States and the United Kingdom, with significantly higher rates in primiparous women [8,9,10]. Approximately 40% of injured women develop symptoms despite primary surgical repair [11].

Surgery for anorectal disorders is another common cause of FI. Multiple surgical procedures have been linked to FI, including internal sphincterotomy and fistulotomy, hemorrhoidectomy, transanal advancement flaps, and internal sphincter dilation with a retractor [12].

Three-dimensional endoanal ultrasound (3D EAUS) is recommended by the International Consultation on Incontinence (ICI) and the European Federation of Societies for Ultrasound in Medicine and Biology (EFSUMB) as the gold-standard imaging technique for the assessment of anal sphincter integrity [13,14]. EAUS has by far the highest resolution, both spatial and temporal, with visualization of submillimeter structures and real-time scanning. The ultrasound anatomy of the anal canal is dominated by the change in arrangement of the muscular layers of the wall, which is made up solely of the concentric muscles of the internal (IAS) and external anal sphincter (EAS). Defects in the IAS are defined as hyperechoic disruptions within the internal anal sphincter ring, and defects in the external anal sphincter are defined as hypoechoic disruptions in the external anal sphincter ring. Although the sonographic diagnosis of anal sphincter injury remains a domain of EAUS, the accuracy of the exam may be hampered by regional acoustic effects, such as intravaginal air. The examination of the sphincters by transperineal ultrasound (TPUS) may provide some additional information, especially in women, but is limited by the diagonal angle of view. The technique is easy and noninvasive; however, the procedure is not yet very popular. Several reports published in the last decade demonstrated a significant correlation between 3D EAUS and transperineal ultrasound (TPUS) findings when used for the detection of obstetric anal sphincter injury (OASI) [15,16]. These studies used mainly high-resolution wide-sector probes and volume imaging commonly applied in obstetric ultrasound practice, but not by gastroenterologists and surgeons. Moreover, these studies were limited to the female population with OASI.

According to the EFSUMB recommendations for gastrointestinal ultrasound [14], TPUS should be performed using a conventional convex 3–5 MHz probe or high-frequency (HF) linear probes (7–15 or 4–9 MHz). The HF probes can provide accurate imaging of the superficial structures in the perianal region. This ultrasound method differs significantly (different ultrasound techniques and equipment) from the high-resolution wide-sector probes and volume imaging used by obstetricians.

3D EAUS provides a right-angled and orthogonal picture of the sphincters [17], while TPUS provides an axial and transversal picture of the sphincters. Therefore, we hypothesized that combining 

3D EAUS and PUS would allow a combined ultrasonographic overview of the anal canal musculature, thereby improving the accuracy of detection of anal sphincter injury.

## 2. Methods

### 2.1. Study Population

We prospectively included in the study all adult patients (>18 years) who underwent ultrasonographic assessment of the anal muscles due to fecal incontinence between January 2020 and January 2021. Our pelvic floor unit serves as a tertiary referral center for the assessment and treatment of FI. All patients undergo a 3D EAUS as part of their regular evaluation.

In order to include only patients with significant symptoms, we encompassed in the fecal incontinence study group only patients who had clinically significant fecal incontinence (defined as solid or liquid fecal incontinence episodes with a frequency of more than once a month). All patients underwent 3D EAUS, followed by TPUS during the same session.

### 2.2. Ultrasound Assessment of the Anal Sphincter

All ultrasound exams were performed using a BK3000 ultrasound machine (Bruel-Kjaer Medical, Peabody, MA, USA), with patients in a left lateral position. Patients were examined by one of two expert clinicians (D.C. or T.E.) with a minimum of 5 years of experience performing 3D EAUS and TPUS and >250 ultrasound exams per annum. All ultrasound images (including three-dimensional volume datasets and TPUS images) were digitized and blindly analyzed retrospectively by the two specialized operators without knowledge of the diagnosis. The presence or absence of sphincter defects was recorded in each imaging method. A final diagnosis of anal sphincter defect was based on the results of both ultrasound methods. Discordant results were reanalyzed by the two ultrasonographers for a final consensus on the presence or absence of defects.

### 2.3. 3D EAUS

**Figure 1:**
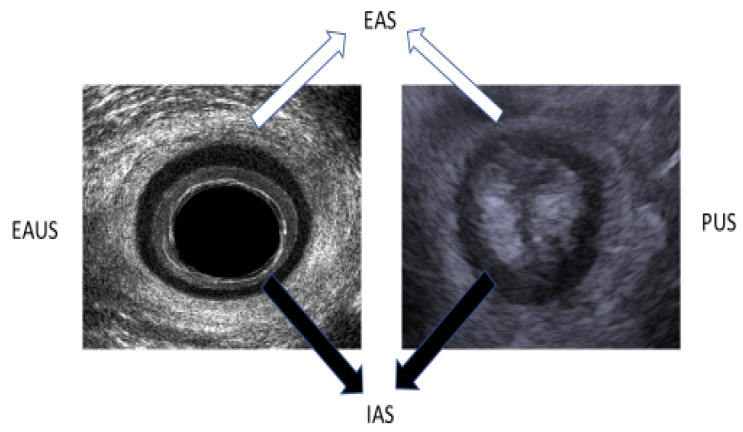
Mid-anal canal image in EAUS (left image) and axial view on PUS (right image). EAS—external anal sphincter. IAS—internal anal sphincter.

**Figure 2:**
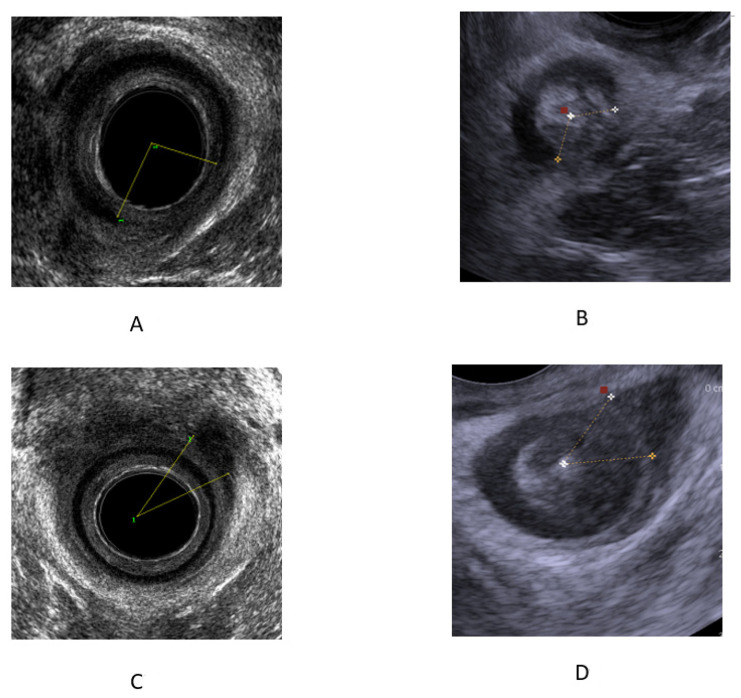
Internal and external anal canal sphincter tear in EAUS and PUS. (**A**) EAUS-IAS tear (**B**) PUS-IAS tear. (**C**) EAUS-EAS tear (**D**) PUS-EAS tear. IAS—internal anal sphincter. EAS—external anal sphincter.

The examination was performed with a transrectal ultrasound using a 360° endoanal probe with three-dimensional (3D) anorectal reconstruction (BK Ultrasound, Peabody, MA, USA) after the performance of a cleansing enema. The examination was performed while the patient was lying in the left lateral position at rest. The ultrasound probe was inserted to the rectum at a depth of 10 cm, and then withdrawn to the level of the anal canal, where the examination of the anal muscular apparatus integrity was performed. Afterward, a 3D reconstruction of the anal canal was performed automatically. The integrity of the puborectalis muscle was measured in the upper anal canal, whereas that of the internal anal sphincter (IAS) and the external anal sphincter (EAS) was measured in the mid-anal canal (Figure 1). Defects in the IAS were defined as hyperechoic (white/gray) echo structures in the internal anal sphincter ring. Defects in the external anal sphincter were defined as hypoechoic (black) echo structures in the external anal sphincter ring (Figure 2).

### 2.4. TPUS

TPUS was conducted using 5–7.5 MHz end-fire transducer or curvilinear 3.5–6 MHz (B&K, 3000, Peabody, MA, USA) probes after liberal application of acoustic gel to the perineum. The examination was performed while the patient was lying in the left lateral position at rest. The examination was performed with the transducer initially applied transversely to the perineal body or the perianal area in order to identify the axial view of the anus using as a landmark the hypo-echoic ring of the internal anal sphincter (Figure 1). The transducer was then turned 180° to obtain a sagittal view of the rectum with extension of the hypoechoic internal anal sphincter appearing above and below the anal canal in profile. The anorectal junction was clearly seen with the bright hyperechoic elliptical bundle of the puborectalis sling. Defects in the internal anal sphincter were defined as hyperechoic (white/gray) echo structures in the internal anal sphincter ring. Defects in the external anal sphincter were defined as hypoechoic (black) echo structures in the external anal sphincter ring (Figure 2).

### 2.5. Statistical Analysis

Dichotomous variables were compared using the chi-square and Fisher’s exact tests where appropriate. A *p*-value < 0.05 was considered significant. Comparisons of objective measurements and detection of anal muscle defects between 3D EAUS and TPUS were reported as sensitivity and specificity. Kappa statistics were used for the determination of inter-test agreement and inter-observer agreement. The 3D EAUS is considered the gold standard for the detection of muscle defects. The strength of agreement was graded as follows: 0–0.20, poor; 0.21–0.40, fair; 0.41–0.60, moderate; 0.61–0.80, substantial; 0.81–1.00, almost complete to complete.

The study was approved by the Sheba Medical Center ethics committee (SMC-20-7840).

## 3. Results

During the period of the study, 108 patients (106 women and two men, mean age 69 ± 13 years) who met the inclusion criteria underwent ultrasonographic assessment of the anal muscle integrity using 3D EAUS and TPUS due to FI. Table 1 summarizes the demographic characteristics of the study population. Inter-observer agreement for the diagnosis of a tear on EAUS and TPUS was high (83%) with Cohen’s kappa of 0.62.

EAUS confirmed anal muscle defects in 53 patients (49%), while TPUS confirmed anal muscle defects in 59 patients (55%). Considering EAUS as the gold standard, the sensitivity and specificity of TPUS for the detection of muscle defects were 82% and 96%, respectively. Cohen’s kappa coefficient was 0.777.

**Figure 3:**
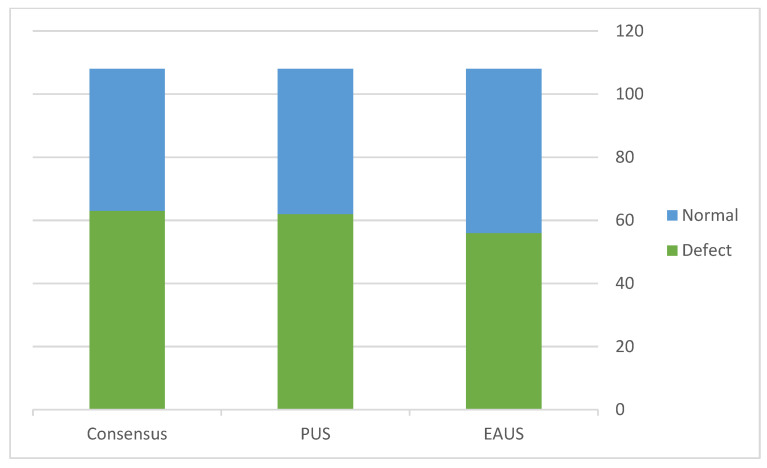
Comparison of the detection of anal muscular defects between different imaging modalities and the final consensus.

After discussing the discordant cases, the final consensus identified the diagnosis of 61 (56%) muscular defects and 47 (44%) normal exams (Figure 3). Therefore, combining the two imaging modalities enabled the detection of muscle defects in 10 cases (16%) missed by the sole use of 3D EAUS and two cases (3%) missed by TPUS. Table 2 summarizes the comparison of 3D EAUS results to that of the final consensus.

## 4. Discussion

To the best of our knowledge, this is the first study to evaluate the combined use of 3D EAUS and TPUS for the detection of anal muscle injury. Most studies to date examined the accuracy of a single ultrasound method for the detection of anal tears. We found that the combination of both techniques improved the detection of anal muscular defects, as both techniques provide different images due to different angles of image uptake.

The precise detection of anal muscle injury is crucial for the workup of FI. The presence of muscular defects, as well as the severity of the defect, may influence treatment decisions and long-term prognosis [18,19]. To date, several methods have been used to evaluate anal sphincter integrity and function, such as digital rectal examination, magnetic resonance, or anal manometry. Above all, EAUS is considered the current gold standard and a safe method for assessing sphincter defects.

The fact that performance of 3D EAUS necessitates special US equipment and software for 3D reconstruction, as well as performance expertise, may limit its use. Therefore, the interest in using transperineal ultrasound for the evaluation of anal sphincter integrity has risen recently. This method does not require special ultrasound equipment, but a significant level of expertise is needed to perform TPUS, and the learning curve for this method is yet unknown. Most studies performed to date concentrated on the accuracy of TPUS (PUS and 3D PUS) for the detection of anal muscular defects through comparison with 3D EAUS [20,21,22,23,24,25,26]. The conflicting results of the studies point to the fact that each imaging modality has inherited strengths and weaknesses. For example, 3D EAUS may fail to detect proximal muscular defects and may demonstrate inconclusive imaging of the anterior mid-anal wall due to disruption of the visualization as a result of the accumulation of air in the vagina. TPUS is limited by the diagonal angle of view and may miss distal pathologies. Assessment of the anal apparatus by TPUS is usually performed through the perineal body in women. However, in men, the quality of the image may be hampered by the disruptive effect of the prostate. Moreover, the comparison of different ultrasound methods may be challenging due to the different visual angles associated with the use of each technique. As the striated fibers of the external anal sphincter stretch in different directions, an erroneous diagnosis of muscular defect can occur [26]. Furthermore, 3D TPUS requires special and expensive ultrasound machines, as well as high levels of expertise, and it is performed mainly by obstetricians and gynecologists.

In this study, we demonstrated that the combined use of 3D EAUS and TPUS significantly enhances the diagnosis of anal muscle defects. TPUS substantially increased the final diagnosis of defects by consensus, although false-positive cases were also noted. The fact that 3D EAUS missed tears in the IAS, which is located in the proximal and middle anal canal, and that TPUS missed tears in the EAS, which is located in the distal anal canal, may be related to the different angle of view of each transducer. A view of the anterior anal wall distorted by artefacts may also partially explain this result.

The performance of TPUS requires the use of micro convex, linear, or end-fire transducers, which are very accessible and affordable, and which can be easily fitted to the US machine with endoanal capabilities. From our experience, the prolongation of the exam time due to the addition of PUS to the 3D EAUS study is neglectable. Although some expertise and experience are required for the performance of TPUS, the beneficial effects of utilizing these imaging methods make it worthwhile for every proctologic and pelvic floor unit.

Our study had limitations. First, the examinations and interpretations were performed by experienced operators using state-of-the-art ultrasonographic equipment. Less experienced examiners might not have obtained the same results. However, it was previously demonstrated that adequate image interpretation of TPUS images was achieved by a less experienced observer [27], although the learning curve of image acquisition and TPUS interpretation has not been examined or defined to date.

Second, we did not compare the angle of the muscular defects between 3D EAUS and PUS, since it has been previously reported that the angles of defect detected by PUS were narrower than those detected by 3D-EAUS due to the difference in image acquisition [28].

The strengths of the study relate to the large cohort and the fact that the entire cohort went through the same ultrasonographic evaluation. Another strength is that all images were reviewed by two experienced ultrasonographers with a high interobserver reliability.

## 5. Conclusions

We found that a combined use of 3D EAUS and PUS during the same exam improved the detection of anal muscular defects. The application of both techniques for the assessment of the anal apparatus should be considered in every patient going through ultrasonographic assessment for anal muscular injury. A better understanding of rhe mechanism responsible for FI might allow better treatment choices for patients with FI.

## Figures and Tables

**Table 1:** Demographic characteristics of the study cohort.

		STD
Age (years) (Mean)	69	13
Female/male	106/2	
Ethnicity White Caucasians	100%	
Deliveries (mean)	2.6	1.7
Cesarean section (% of all deliveries)	14 (5%)	
Assisted deliveries (% of all deliveries)	10 (3.6%)	
Previous anorectal surgery (% of the study cohort)	28 (26%)	

Deliveries are presented as the mean. Cesarean sections and assisted deliveries are presented as the total and percentage of all deliveries. Previous anorectal surgery is presented as the total and percentage of the total cohort.

**Table 2:** Comparison of 3D EAUS and TPUS to the final consensus.

	Consensus	3-D EAUS	TPUS
Defects	61	53	59
EAS	38	36	38
IAS	19	13	18
EAS + IAS	4	4	4
Normal	47	56	49
Sensitivity		0.86	0.96
Specificity		1	1

The results of the consensus on the diagnosis of anal tear versus the diagnosis of tear in each technique (3D EAUS and TPUS) are presented in this table. EAS—external anal sphincter; IAS—internal anal sphincter.

## Data Availability

Data can be available at request.
